# Physical activity, memory function, and hippocampal volume in adults with Down syndrome

**DOI:** 10.3389/fnint.2022.919711

**Published:** 2022-09-13

**Authors:** Jamie C. Peven, Benjamin L. Handen, Charles M. Laymon, Victoria Fleming, Brianna Piro-Gambetti, Bradley T. Christian, William Klunk, Ann D. Cohen, Ozioma Okonkwo, Sigan L. Hartley

**Affiliations:** ^1^Department of Psychiatry, University of Pittsburgh, Pittsburgh, PA, United States; ^2^Department of Radiology and Bioengineering, University of Pittsburgh, Pittsburgh, PA, United States; ^3^School of Human Ecology, University of Wisconsin, Madison, WI, United States; ^4^Waisman Center, University of Wisconsin, Madison, WI, United States; ^5^Department of Medical Physics, University of Wisconsin, Madison, WI, United States; ^6^Department of Medicine, University of Wisconsin, Madison, WI, United States

**Keywords:** Down syndrome, physical activity, memory, hippocampus, cognition

## Abstract

Higher engagement in moderate-intensity physical activity (PA) is related to better cognitive functioning in neurotypical adults; however, little is known about the effect of PA on cognitive aging in adults with Down syndrome (DS). Individuals with DS have three copies of chromosome 21, which includes the gene involved in the production of the amyloid precursor protein, resulting in an increased risk for an earlier onset of Alzheimer’s disease (AD). The goal of this study was to understand the relationship between engagement in moderate PA, memory, and hippocampal volume in adults with DS. Adults with DS participated in an ancillary Lifestyle study linked to the Alzheimer’s Biomarkers Consortium for DS (ABC- DS; *N* = 71). A within-sample z-score memory composite was created from performance on the Cued Recall Test (CRT) and the Rivermead Picture Recognition Test. Participants wore a wrist-worn accelerometer (GT9X) to measure PA. Variables of interest included the average percentage of time spent in moderate PA and average daily steps. Structural MRI data were acquired within 18 months of actigraphy/cognitive data collection for a subset of participants (*n* = 54). Hippocampal volume was extracted using Freesurfer v5.3. Associations between moderate PA engagement, memory, and hippocampal volume were evaluated with hierarchical linear regressions controlling for relevant covariates [age, body mass index, intellectual disability level, sex, and intracranial volume]. Participants were 37.77 years old (SD = 8.21) and were 55.6% female. They spent 11.1% of their time engaged in moderate PA (SD = 7.5%) and took an average of 12,096.51 daily steps (SD = 4,315.66). After controlling for relevant covariates, higher memory composite score was associated with greater moderate PA engagement (*β* = 0.232, *p* = 0.027) and more daily steps (*β* = 0.209, *p* = 0.037). In a subset of participants, after controlling for relevant covariates, PA variables were not significantly associated with the hippocampal volume (all *p*-values ≥ 0.42). Greater hippocampal volume was associated with higher memory composite score after controlling for relevant covariates (*β* = 0.316, *p* = 0.017). More PA engagement was related to better memory function in adults with DS. While greater hippocampal volume was related to better memory performance, it was not associated with PA. Greater PA engagement may be a promising lifestyle behavior to preserve memory in adults with DS.

## Introduction

Greater engagement in physical activity (PA), particularly PA of at least moderate intensity, is associated with less decline in memory functioning with age (Voss et al., 2019) and reduced risk of dementia in neurotypical adults (Blondell et al., 2014; Xu et al., 2017). These benefits are thought to be driven by the effect of engagement in PA on brain health; greater PA engagement is related to less age-related reduction in regional brain volumes (Hillman et al., 2008; Erickson et al., 2014), including hippocampal volume. These findings have led to clinical trials of PA interventions aimed at fostering healthy brain and cognitive aging and reducing the risk for Alzheimer’s disease (AD) in the general population, with mixed results (Etgen et al., 2010; Morris et al., 2017; Lamb et al., 2018; Erickson et al., 2019a). Adults with Down syndrome (DS) are genetically at increased risk of AD with the onset of clinical symptoms reported to range from the mid-40s to late-60s (Holland et al., 2000; McCarron et al., 2017). Individuals with DS tend to maintain lifestyles with low PA engagement compared to the neurotypical population (Sundahl et al., 2016; Oreskovic et al., 2020) due to a variety of reasons including environmental factors (e.g., lack of social support, activities that aren’t fun) or health factors (e.g., chronotropic incompetence and being overweight/obese; Mahy et al., 2010; Bossink et al., 2017). It remains unknown whether differences in the level of PA engagement among adults with DS are associated with early memory decline or with biomarkers of brain pathology associated with aging and AD, including reduced hippocampal volume.

DS is a genetic disorder caused by full or partial trisomy 21 and has an incidence of 1 in 691 live births in the US (Mai et al., 2019) and 1 in 1,000–1,100 worldwide (World Health Organization, 2018). The triplication of the amyloid precursor protein gene, found on chromosome 21, is hypothesized to cause overexpression of amyloid-β which leads to amyloid-β plaques in the brain by the fifth decade of life (Mann et al., 1990; Annus et al., 2016)—an early feature of the neuropathology characteristic of Alzheimer’s disease. The accumulation of amyloid-β plaques is followed by the development of neurofibrillary tangles of the protein tau and neurodegeneration, including a reduction in hippocampal brain volume, a region of importance for memory functioning (Squire, 2004; Opitz, 2014). Biomarkers of hippocampal volume reduction are evident in adults with DS prior to the onset of AD clinical dementia (Beacher et al., 2009; Lott and Head, 2019; Fortea et al., 2021), which has a mean age of onset in the mid-50s in DS (Holland et al., 2000; McCarron et al., 2017) and occurs more than 20 years earlier than sporadic late-onset AD in the neurotypical adult population (Teipel and Hampel, 2006; Wilcock and Griffin, 2013; Neale et al., 2018). In neurotypical adults, reduced hippocampal volume is closely related to poorer memory function, particularly later in life (Opitz, 2014; Pohlack et al., 2014; de Flores et al., 2015). However, this relationship has not yet been established in adults with DS who may not show signs of AD clinical dementia but do have evidence of reduced hippocampal volume.

To date, limited research has investigated the relationship between PA and memory function or hippocampal volume in the DS population. Mouse models demonstrate that aerobic exercise promotes hippocampal neurogenesis (Liu and Nusslock, 2018; Kim et al., 2019) and improves performance on spatial memory tasks (Mustroph et al., 2012; Xiong et al., 2015). These findings have been extended to mouse models of DS (Llorens-Martín et al., 2010; Parrini et al., 2017). In neurotypical adults, greater engagement in PA is associated with larger hippocampal volume (Aghjayan et al., 2021; Machida et al., 2021), better memory function (Hayes et al., 2015), and better maintenance of both hippocampal volume and memory function over time (Erickson et al., 2011; Wilckens et al., 2021). In adults with DS, studies have reported mixed findings regarding the benefit of acute exercise on cognitive functioning, with evidence of improvements in inhibitory control and processing speed (Chen and Ringenbach, 2016) but not other areas of cognition. Results from a clinical trial of an exercise intervention program also reported improvements in memory in adults with DS (aged 18–35 years) after 12 weeks of one or two 30-min exercise sessions per week (Ptomey et al., 2018). Cross-sectional findings from our group also found that adults with DS who spent more time engaged in moderate PA in their everyday lives performed better on measures of verbal response inhibition, visual-motor integration, and global cognitive functioning compared with more sedentary individuals (Fleming et al., 2021). Thus, there is a need to investigate the associations between PA engagement in adults with DS, memory function, and hippocampal volume.

The goal of the present study was to understand the relationship between PA engagement, memory function, and hippocampal volume in a sample of 71 young and middle-aged adults with DS. We predicted that greater PA engagement would be associated with both better memory function and larger hippocampal volume. Further, we predicted that larger hippocampal volume would be associated with better memory performance, regardless of PA engagement.

## Materials and Methods

### Participants

Eighty participants were recruited from the longitudinal Alzheimer’s Biomarkers Consortium for Down Syndrome (ABC-DS^1^), a longitudinal research study aimed at identifying early biomarkers of AD in DS. Participants from the University of Wisconsin-Madison and University of Pittsburgh sites were invited to enroll in an ancillary study examining the effect of Lifestyle factors on AD biomarkers (R01AG070028; PI: Hartley). Inclusion criteria included age ≥25 years, having a mental age ≥30 months, and genetic testing confirming DS (full trisomy, partial trisomy, or mosaic). Exclusion criteria included contraindications to neuroimaging (e.g., pregnant or metallic implants), and medical or mental health conditions that affected cognitive functioning. Written informed consent was obtained for the adult with DS or their legal guardian, with assent obtained from the adult with DS if the latter. The study was approved by the University of Wisconsin-Madison and University of Pittsburgh Institutional Review Boards in accordance with the Declaration of Helsinki.

### Procedures

Adults with DS completed a 2-day study visit to either the University of Wisconsin-Madison or the University of Pittsburgh to complete a neuropsychological battery and neuroimaging scans (see Handen et al., 2020 for details on the larger study protocol). A caregiver also attended the study visit and completed interviews and questionnaires regarding the adult with DS’s health history, cognition, functioning, and behavior. The adult with DS was then equipped with a wrist-worn accelerometer, an activity-monitoring device, for seven consecutive days to assess PA in everyday life. The adult with DS and their caregiver also completed an activity diary across these 7 days to aid in the interpretation of the accelerometer data. Structural magnetic resonance imaging (MRI) was used to obtain total intracranial volume (ICV) and hippocampal volume estimates. Data collection for PA, memory, and hippocampal volume took place over an average of 8.6 months (range = 0–18; *n* = 54). MRI data that were collected more than 18 months before or after cognitive and PA data were excluded because the relationship between PA and brain structure has been shown to be time sensitive through exercise intervention research in neurotypical populations (Tamura et al., 2015; Wilckens et al., 2021). Since ABC-DS study visits occur in 16-month cycles, neuroimaging data used for the current study were collected within one cycle of the cognitive and PA data to ensure that any conclusions drawn about hippocampal volume were temporally correlated with cognition and PA engagement.

### Measures

#### Demographic information

Chronological age was calculated using the date of birth and the date of study visit. Body mass index (BMI) was calculated as weight in kilograms (kg) divided by height in meters squared (m^2^; kg/m^2^). Additional medical information was collected regarding the presence of hypothyroidism, cardiovascular conditions, and obstructive sleep apnea (OSA). These conditions were selected due to higher rates of the conditions in the DS population (Versacci et al., 2018; Rafii et al., 2019; Antonarakis et al., 2020), and their associations with lower PA engagement (Biswas et al., 2015; Van Offenwert et al., 2019). Overall cognitive mental age was determined based on performance on the Peabody Picture Vocabulary Test – 4th Edition (PPVT-4; Dunn and Dunn, 2007) at ABC-DS study entry (prior to clinical dementia) and used to estimate the level of intellectual disability. The PPVT-4 has good reliability and validity in adults with DS (Phillips et al., 2014; Fleming et al., 2021). Mental age equivalents of 9 years were considered a mild intellectual disability, those of 6–8 years were considered a moderate intellectual disability, and those of 3–6 years were considered severe a intellectual disability.

#### Clinical dementia status

Clinical dementia status was determined based on a case consensus conference that involved a licensed psychologist, physician, and staff with expertise in AD in DS who were blind to PA and neuroimaging data. Details about the consensus process have been previously published (Handen et al., 2020). The clinical dementia status of the adult with DS was determined from review of caregiver reported information on cognition, adaptive functioning, and behavior, and directly administered measures of cognitive functioning, and in consideration of the level of intellectual disability, psychiatric and medical conditions, and recent major life events. Participants were classified into one of the following clinical dementia statuses: (1) cognitively stable, meaning that there was no evidence of cognitive decline beyond what would be expected in normative aging; (2) mild cognitive impairment—DS (MCI-DS), indicating subtle and/or limited decline in cognition and/or adaptive behavior; (3) AD, indicating marked declines in cognition and adaptive behavior, or (4) unable to determine, meaning that that dementia status was not clear due to recent life events or changes in medical or psychiatric conditions.

#### Physical activity

Participants wore a GT9X accelerometer (Actigraph LLC, Pensacola, FL) on their non-dominant wrist for 7 days to objectively monitor habitual PA. Participants were asked to wear the actigraph at all times, including during sleep, except during long-duration water-based activities (e.g., swimming). Data from the actigraph watches were analyzed using ActiLife software (version 6.13.4) and Troiano algorithms (Troiano et al., 2008). Freedson adult cut points were used to classify each minute of PA into different intensity levels based on a uniaxial algorithm of metabolic counts per minute (Freedson et al., 1998): sedentary (<99), light (100–1,951), moderate (1,952–5,724), and vigorous (>5,725). Although these cut points were initially developed on hip-worn accelerometers, they have been used in wrist-worn accelerometers and capture differences in physical activity in free living (Rowlands and Stiles, 2012; Mannini et al., 2013). Wrist-worn devices have also been found to have better compliance compared to hip-worn accelerometers (Troiano et al., 2014; Montoye et al., 2020). These intensity levels correspond to engagement in different types of activities. For example, reaching a moderate PA level would consist of taking a brisk walk, while reaching a light PA level may consist of standing to prepare food. Only participants with at least 4 days and at least 12 h of data per day were included in the current study. Data collected on days with less than 12 h of data were removed to better gauge habitual activity without the confound of limited actigraph wear-time (Zabetian-Targhi et al., 2021; 83%) of the participants wore the actigraph for 12 daytime hours/day on four or more days, while seven (10%) wore it for at least 10 daytime hours and five (7%) for at least nine daytime hours. The average daily level of PA was calculated and used in analyses. Variables of interest included the percentage of time engaged in moderate-intensity activity and daily step count, as these reflect intentional exercise and habitual activity throughout the day, respectively, and have been associated with the hippocampal volume and memory function (Hayes et al., 2015; Makizako et al., 2015; Machida et al., 2021; Zabetian-Targhi et al., 2021). The percentage of time engaged in moderate PA was calculated by dividing the number of minutes engaged in moderate PA by the total number of minutes the actigraph watch was worn each day.

#### Memory function

Episodic memory was evaluated through recall and recognition tests. The **modified Cued Recall Test** (CRT; Zimmerli and Devenney, 1995) asks participants to learn three sets of four pictures and to recall the names of those pictures immediately after learning all three sets. Recall trials are initially administered as the items that can be freely recalled; when participants cannot freely recall additional items, they are given a standardized cue for any unnamed items. Three recall trials are administered. The total number of correctly recalled items (free + cued) across all three trials was used as the CRT outcome measure. The ***Rivermead Picture Recognition Test*** (RPR; de Wall et al., 1994) asks participants to name 10 pictures (each shown once for 5 s) and to recognize them after a delay from a set of 20 randomly presented pictures (comprising the 10 previously shown pictures and 10 distractors). The total number of hits plus true negatives identified during the delayed recognition trial was used as the RPR outcome measure.

Similar to Bazydlo et al. (2021), a memory composite score based on the CRT and RPR was created for statistical analyses. Within-sample z-scores were calculated for CRT total recall and RPR total scores. Then, the z-scores were averaged to create a memory composite score. Both the CRT and RPR have been shown to have good test properties in adults with DS (Zimmerli and Devenney, 1995; Mihaila et al., 2019). Both tests have also been found to be sensitive to early AD-related biomarker changes in adults with DS prior to the onset of clinical dementia (Hartley et al., 2014, 2017).

#### Hippocampal volume

##### Acquisition

Participants underwent whole-brain structural MRI on a 3T GE Discovery MR750 scanner at the University of Wisconsin-Madison or a 3T Siemens Prisma scanner at the University of Pittsburgh. High-resolution T1-weighted brain images were acquired using a 3-dimensional fast spoiled gradient echo (FSPGR) sequence at the University of Wisconsin-Madison or a magnetization prepared rapid-acquisition gradient echo (MPRAGE) sequence at the University of Pittsburgh, consistent with the Alzheimer’s Disease Neuroimaging Initiative 3 (ADNI3) and Human Connectome Project protocols. Sagittal images were acquired at both the University of Wisconsin-Madison (slice thickness = 1.2 mm, repetition time = 7.35 ms, echo time = 3.04 ms, matrix = 256 × 256 × 196 pixels, flip angle = 11°) and the University of Pittsburgh (slice thickness = 1.2 mm, repetition time = 2,300.0 ms, echo time = 2.95 ms, matrix = 240 × 256 × 176 pixels, flip angle = 9°). The hippocampal volume data for a subset of 54 participants were used in analyses for the current study.

##### Processing

Neuroimaging data were processed at the Mayo Clinic Aging and Dementia Imaging Lab with Freesurfer^2^ version 5.3 and the Desikan-Killany atlas (Desikan et al., 2006). Hippocampal volume was parsed into left and right volumes (in mm^3^), and then summed for total hippocampal volume, which was used for this study.

### Statistical analyses

Statistical analyses were completed using IBM SPSS version 28.0.0 (IBM Corporation, 2020) and R Studio version 2021.09.1 (R Team, 2020). Descriptive statistics, box plots, and histograms were used to examine the distribution of main study variables and identify any outliers. Independent sample t-tests and chi-square statistics were conducted to determine any differences in demographic information between the adults with DS who had valid actigraph and MRI data vs. those without. Bivariate Pearson’s product moment correlations examined the association between demographic characteristics (i.e., chronological age, BMI, intellectual disability level, sex, hypothyroidism, cardiovascular disease, and OSA), PA variables (i.e., the average percentage of time engaged in moderate activity and average daily steps), memory composite score, and total hippocampal volume in order to determine relevant covariates for analyses of interest. The following demographic characteristics were significantly related to PA, memory, or hippocampal volume and controlled for in analyses: chronological age, intellectual disability level, and sex. Associations between PA variables, memory function, and hippocampal volume were assessed using hierarchical linear regressions that included the relevant demographic controls (i.e., those significantly related to main study variables), as well as intracranial volume when appropriate, in two levels: (1) relevant covariates, and (2) variables of interest (i.e., average moderate PA time, average daily steps). Data were confirmed to meet the assumptions of linear regression prior to interpretation.

## Results

### Participants

Of the 80 adults with DS enrolled in the Lifestyle study, 71 successfully wore the actigraph watch for at least 4 days and had at least 12 h of valid daytime data collection each day. The nine excluded participants did not differ from the 71 participants with valid actigraphy data on any demographic characteristics except sex, where only 22% of excluded participants were female (*p* < 0.001). On average, included participants were 37.77 years old (SD = 8.21 years), 56% were female, and 100% identified as White. The average BMI was above 33 kg/m^2^ (33.46 ± 8.16 kg/m^2^), indicating that most participants met the criteria for obesity. Four participants had a clinical status of MCI-DS (6%), one had a clinical status of dementia (1%), two had a clinical status of unable to determine (3%), and 64 were cognitively stable (90%). Forty participants had mild intellectual disability (56%), 20 had moderate intellectual disability (28%), and 11 had severe intellectual disability (15%). See Table 1 for additional demographic details.

Of the 71 participants with valid PA data, 54 had MRI data that were acquired within 18 months of the cognitive and PA data acquisition (17 had MRI data that were out of the 18-month range). Analyses involving hippocampal volume therefore only included this subset of 54 participants. Independent sample t-tests and chi-square statistics indicated that there were no significant differences in demographic characteristics, PA engagement, or memory function between the 54 participants with MRI data and the remaining adults with DS enrolled in the Lifestyle study (all *t*-values < |0.40|, all *p*-values > 0.69).

**Table 1:** Participant demographic characteristics (*N* = 71).

**Age (years), M ± SD**	37.7 ± 8.21
**Sex, %**	
Female	55.6%
**Race, %**	
White	100%
**Body Mass Index** (kg/m^2^), M ± SD	33.46 ± 8.16
**Intellectual Disability Level**	
*Mild*	56.3%
*Moderate*	28.2%
*Severe*	15.5%
**Obstructive Sleep Apnea**	
*Present*	46.5%
*Treated*	57.6%
**Cardiovascular Condition Presence**	31.0%

*Note: M, mean; SD, standard deviation; kg, kilograms; m, meters*.

### Physical activity engagement

**Figure 1:**
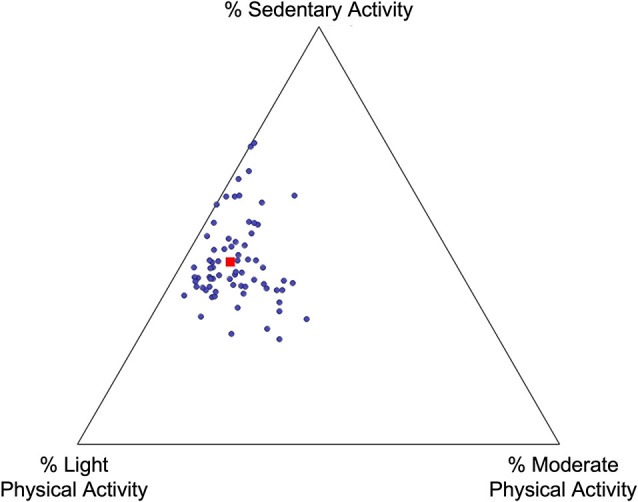
Physical activity distribution. Each point represents one participant; their location on the plot represents the proportion of time spent engaged in moderate physical activity, light physical activity, and sedentary activity. The red square represents the mean breakdown of time across all participants.

On average, participants spent 11% of their daily time engaged in a moderate-intensity activity (11.14 ± 7.49%). They took an average of 12,096.51 steps per day (SD= 4,315.66 steps). Participants spent almost 44% of their time engaged in sedentary behavior (43.54 ± 9.93%), including sitting, laying, or sleeping. The remaining time during the day was spent engaged in a light-intensity activity (45.37 ± 8.09%), such as leisurely walking or body moving. No participant engaged in activity in the vigorous-intensity range. See Table 2 and Figure 1 for additional PA details. Figure 1 depicts the proportion of time each participant spent engaging in moderate PA, light PA, or sedentary activities. For example, dot location closer to the “Light PA” vertex would indicate more time engaged in light PA relative to moderate PA or sedentary activities.

**Table 2:** Physical activity characteristics (*N* = 71).

	**Mean**	**Standard deviation**	**Minimum**	**Maximum**
Sedentary (%)	43.54	9.93	25.18	72.17
Light PA (%)	45.37	8.09	25.20	70.08
Moderate PA (%)	11.14	7.49	0.22	32.53
Daily Steps	12,096.51	4,315.66	3,333.00	20,456.29

*Note: PA, physical activity*.

### Memory function

On the CRT, participants recalled an average of 31.93 total items on the CRT (SD = 6.81, range = 7–36 items). Total items correctly recognized on the RPR was 15.87 (SD = 4.57, range = 0–20). CRT and RPR raw scores were converted to within-sample z-scores and then averaged to create the memory composite score. Memory composite z-scores ranged from 2.47 to 0.75 with an SD of 0.84.

### Hippocampal volume

Of the 54 participants with MRI data, the average total hippocampal volume was 6,869.39 ± 1,179.34 mm^3^. There was no significant difference between left (3,386.87 ± 611.22 mm^3^) and right (3,482.53 ± 597.67 mm^3^) hippocampal volumes. Average ICV was 1,230,877.29 mm^3^ (SD = 161,042.92 mm^3^).

### Relevant covariates

We identified demographic variables (i.e., chronological age, sex, BMI, intellectual disability level, presence of OSA, presence of cardiovascular conditions) associated with PA variables of interest (i.e., average percentage of time engaged in moderate activity and average daily step count), memory, and hippocampal volume. Only chronological age was significantly correlated with time spent in moderate PA. Chronological age and intellectual disability level were significantly associated with memory function. Chronological age and sex were significantly associated with the hippocampal volume. BMI was also included as a covariate, despite non-significant associations with our variables of interest, to be certain that any significant effects of PA engagement were not due to the influence of BMI (Gay et al., 2016). ICV was also included as a covariate in all analyses involving hippocampal volume. All bivariate correlation coefficients are shown in Table 3.

**Table 3:** Bivariate correlations between demographic factors, physical activity, memory, and hippocampal volume (subset of *n* = 54).

	Moderate PA	Light PA	Sedentary Time	Daily Steps	Memory Composite Score	Total Hippocampal Volume
Age	−0.31**	0.32**	−0.02	−0.15	−0.27*	−0.34*
Sex	0.03	−0.19	0.13	−0.14	0.22	−0.31*
BMI	−0.01	−0.13	0.06	−0.11	0.21	−0.02
ID level	−0.20	−0.06	0.22	−0.19	−0.50**	−0.07
OSA presence	−0.22	0.06	0.12	−0.13	−0.16	0.06
CC presence	0.11	0.13	−0.19	0.22	0.03	−0.01

Note: BMI, body mass index; ID, intellectual disability; OSA, obstructive sleep apnea; CC, cardiovascular conditions. Sex was coded as 1 = male, 2 = female; ID level was coded as 1 = mild, 2 = moderate, 3 = severe. *indicates *p* < 0.05, **indicates *p* < 0.01.

### Physical activity, memory function, and hippocampal volume

**Figure 2:**
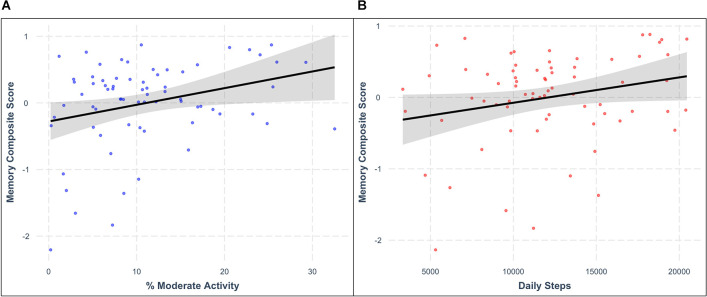
Relationships between memory composite score and **(A)** average percentage of time spent in moderate physical activity (*β* = 0.232, *p* = 0.027) and **(B)** average daily steps (*β* = 0.209, *p* = 0.037) after controlling for relevant covariates. Shaded areas represent the 95% confidence interval for each regression model.

Consistent with our hypotheses, greater PA engagement was related to better memory function. After controlling for chronological age, BMI, and intellectual disability level, higher percentage of time spent in moderate-intensity PA was associated with higher memory composite scores (model *F*_(4,59)_ = 14.653, moderate PA *β* = 0.232, *p* = 0.027; Figure 2A). Similarly, greater average daily steps were associated with better memory composite scores (model *F*_(4,59)_ = 14.382, daily steps *β* = 0.209, *p* = 0.037; Figure 2B) after controlling for the same demographic characteristics. See Table 4 for additional details.

**Table 4:** Regression models of the relationships between physical activity variables and memory composite score (*N* = 71).

Predictor	*β*	*β* 95% CI	*sr* ^2^	*sr*^2^ 95% CI	*r*	Model fit
Age	−0.35	(−0.55, −0.15)	0.09	(−0.01, 0.19)	−0.27*
BMI	0.13	(−0.04, 0.31)	0.02	(−0.03, 0.06)	0.19
ID level	−0.55	(−0.74, −0.35)	0.24	(0.09, 0.40)	−0.50**
Moderate PA	0.23	(0.04, 0.42)	0.04	(−0.03, 0.11)	0.44**
						R^2^ Δ = 0.044*
						*Overall R^2^* = 0.494**
						95% CI (0.29, 0.60)
Age	−0.40	(−0.58, −0.21)	0.13	(0.02, 0.25)	−0.27*
BMI	0.15	(−0.03, 0.33)	0.02	(−0.03, 0.07)	0.19
ID level	−0.56	(−0.76, −0.37)	0.27	(0.11, 0.43)	−0.50**
Daily Steps	0.20	(0.02, 0.39)	0.04	(−0.03, 0.10)	0.36**
						R^2^ Δ = 0.039*
						*Overall R^2^* = 0.488**
						95% CI (0.28, 0.59)

Note: β, standardized regression weights; *sr*^2^, semi-partial correlation squared; *r*, bivariate correlation; BMI, body mass index; ID, intellectual disability. Variables were entered into the regression models in the order presented; level 1 = all covariates, level 2 = individual physical activity variables. *R*^2^ Δ indicates the change from level 1 to level 2. *indicates *p* < 0.05, **indicates *p* < 0.01.

**Figure 3:**
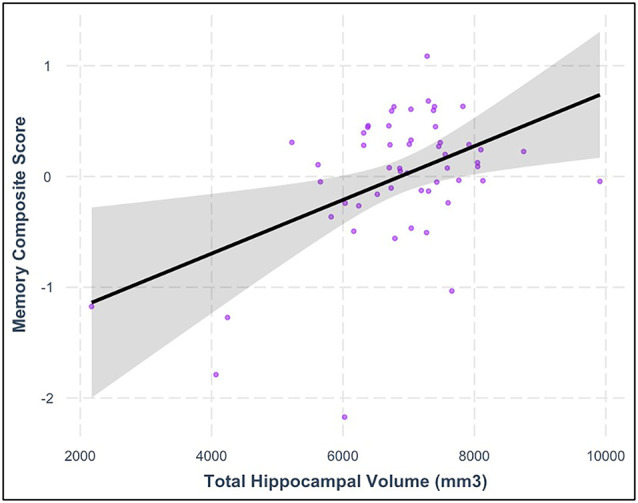
Relationship between total hippocampal volume and memory composite score after controlling for relevant covariates (*β* = 0.316, *p* = 0.017). The shaded area represents the 95% confidence interval for the regression model.

Consistent with our hypotheses, greater total hippocampal volume was associated with higher memory composite scores (model *F*_(6,44)_ = 11.910, hippocampal volume *β* = 0.316, *p* = 0.017) after controlling for chronological age, BMI, intellectual disability, sex, and ICV. See Table 5 and Figure 3 for additional details. In contrast with our hypotheses, neither moderate PA time nor daily steps were significantly related to the hippocampal volume in this sample after controlling for chronological age, BMI sex, and ICV (all models’ *F*_(6,44)_ ≤ 6.683, all PA variables’ *p*-values ≥ 0.42; Table 6).

**Table 5:** Regression models of the relationships between physical activity variables and hippocampal volume (*n* = 54).

Predictor	*β*	*β* 95% CI	*sr* ^2^	*sr*^2^ 95% CI	*r*	Model fit
Age	−0.56	(−0.81, −0.31)	0.22	(0.05, 0.40)	−0.34*
Sex	0.03	(−0.26, 0.33)	0.00	(−0.01, 0.01)	0.31*
BMI	0.16	(−0.08, 0.39)	0.02	(−0.04, 0.08)	−0.02	
ID level	−0.21	(−0.46, 0.04)	0.03	(−0.04, 0.10)	−0.07
Intracranial Volume	0.59	(0.29, 0.90)	0.18	(0.02, 0.34)	0.41**
Moderate PA	0.09	(−0.22, 0.26)	0.00	(−0.01, 0.01)	0.16
						*R^2^ Δ = 0.001*
						*Overall R^2^* = 0.463**
						95% CI (0.18, 0.56)
Age	−0.57	(−0.81, −0.33)	0.25	(0.07, 0.43)	−0.34*
Sex	0.04	(−0.26, 0.33)	0.00	(−0.01, 0.01)	0.31*
BMI	0.15	(−0.09, 0.39)	0.02	(−0.03, 0.07)	−0.02
ID level	−0.22	(−0.47, 0.04)	0.03	(−0.04, 0.11)	−0.07
Intracranial Volume	0.59	(0.29, 0.99)	0.18	(0.02, 0.34)	0.41**
Daily Steps	−0.01	(−0.25, 0.23)	0.00	(−0.00, 0.00)	0.13
						* R^2^Δ = 0.00*
						*Overall R^2^* = 0.463**
						95% CI (0.18, 0.56)

Note: β, standardized regression weights; *sr*^2^, semi-partial correlation squared; *r*, bivariate correlation; BMI, body mass index; ID, intellectual disability. Variables were entered into the regression models in the order presented; level 1 = all covariates, level 2 = individual physical activity variables. *R*^2^ Δ indicates the change from level 1 to level 2. *indicates *p* < 0.05, **indicates *p* < 0.01.

**Table 6:** Regression model of the relationship between total hippocampal volume and memory composite score (*n* = 54).

Predictor	*β*	*β* 95% CI	*sr* ^2^	*sr*^2^ 95% CI	*r*	Model fit
Age	−0.29	(−0.53, −0.04)	0.05	(−0.03, 0.12)	−0.25	
Sex	−0.01	(−0.26, 0.24)	0.00	(−0.00, 0.00)	−0.15	
BMI	−0.21	(0.01, 0.41)	0.04	(−0.03, 0.10)	0.31*	
ID level	−0.59	(−0.80, −0.39)	0.28	(0.11, 0.45)	−0.55**	
Intracranial volume	−0.08	(−0.38, 0.21)	0.00	(−0.01, 0.02)	−0.08	
Hippocampal volume	−0.32	(0.09, 0.58)	0.06	(0.02, 0.14)	0.43**	
						R^2^ Δ = 0.053*
						*Overall R^2^* = 0.615**
						95% CI (0.37, 0.69)

Note: β, standardized regression weights; *sr*^2^, semi-partial correlation squared; *r*, bivariate correlation; BMI, body mass index; ID, intellectual disability. Variables were entered into the regression model in the order presented; level 1 = all covariates, level 2 = hippocampal volume. *R*^2^ Δ indicates the change from level 1 to level 2. *indicates *p* < 0.05, **indicates *p* < 0.01.

## Discussion

We sought to examine the relationships between PA, memory function, and hippocampal volume in adults with DS. Consistent with our first hypothesis, greater engagement in moderate PA was associated with better memory function. The relationship between average daily steps and memory function was also statistically significant after controlling for relevant covariates. In contrast to our second hypothesis, PA and hippocampal volume were not related in our sample of adults with DS. Finally, consistent with our third hypothesis, larger hippocampal volume was associated with better memory function. Together, these findings provide considerable evidence that the cognitive benefits of PA engagement seen in neurotypical populations extend to the DS population, as does the relationship between hippocampal volume and memory function.

To our knowledge, this is the first study to demonstrate an association between greater objectively monitored PA engagement and better memory function in a cross-sectional sample of adults with DS. Even after accounting for chronological age, BMI, and level of intellectual disability, more PA engagement was associated with better memory performance. This suggests that PA may be a key modifiable lifestyle behavior that could bolster memory performance in a population at-risk for early-onset memory decline due to AD. Notably, both our PA variables of interest were significantly associated with memory function and likely mean that greater time spent in moderate-intensity intentional exercise (e.g., swimming or fast walking) may be an important component in interventions to promote healthy aging in DS. PA literature across neurotypical populations highlights the role of at least moderate intensity PA in promoting better cognitive aging (Middleton et al., 2010; Carvalho et al., 2014; Erickson et al., 2019b). Our findings support and extend this literature to non-neurotypical, mid-life adults with DS. Further, we were able to demonstrate that habitual activity, or daily steps, was also associated with memory function regardless of PA intensity. This suggests that being more active, in any sense (e.g., light and moderate-intensity activity), is likely beneficial to cognitive aging in the DS population. While the effect sizes of these relationships were small, they are consistent with PA and cognitive or brain health literature in the neurotypical population (Smith et al., 2010; Wilckens et al., 2021). It is important to consider that this same relationship has not been consistently demonstrated in younger neurotypical populations (Cox et al., 2015; Engeroff et al., 2018; Stern et al., 2019), suggesting that DS memory abilities are potentially more vulnerable at an earlier age compared to the neurotypical population. Given our knowledge that clinical AD develops 20 years earlier in adults with DS than neurotypical adults (Wilcock and Griffin, 2013; Neale et al., 2018), increasing even lower intensity PA engagement may be beneficial to promote healthier cognitive aging. This highlights the importance of occupational and leisure activity, as steps per day are an easy-to-measure marker of one healthy behavior.

Our results further extend the well-studied relationship between hippocampal volume and memory performance in neurotypical adults to those with DS. In aging neurotypical populations, a larger hippocampal volume has been associated with better memory performance across verbal and visual tasks for recall and recognition (de Flores et al., 2015; Lazarov and Hollands, 2016; Bennett et al., 2019). While these findings are often pronounced in older adults, they are present in younger neurotypical populations as well (Pohlack et al., 2014; Bråthen et al., 2018). Here, we show that hippocampal volume and memory performance were closely related in mid-life adults with DS whose hippocampal volume may have already been at a disadvantage compared to the neurotypical population due to trisomy 21 (Pinter et al., 2001). Extending this association to a DS population underscores the role of the hippocampus in memory formation and function, particularly after controlling for the effects of chronological age, sex, intellectual disability, and BMI—demographic factors likely to influence global and regional brain volume.

It is not clear why PA engagement was not related to hippocampal volume in this adult DS sample. It is possible that some of the benefits of PA on memory may occur in other mechanisms in adults with DS such as by increasing growth factor expression (Cotman et al., 2007) or cerebral blood flow (Brown et al., 2010), and improving white matter microstructural integrity (Gons et al., 2013; Aghjayan et al., 2021), as has been shown in neurotypical populations. It is also important to note that physiological differences in DS include a predisposition for congenital heart defects and other cardiovascular-related changes, sleep apnea, and hypothyroidism (Amr, 2018; Versacci et al., 2018; Rafii et al., 2019; Antonarakis et al., 2020), which may preclude individuals with DS from achieving higher intensities of PA in everyday life. Dose-response exercise research in other populations (e.g., stroke, neurotypical older adults) is mixed regarding whether PA intensity is related to brain-derived neurotrophic factor (BDNF) expression (Boyne et al., 2019; Kovacevic et al., 2020), which is associated with hippocampal neurogenesis (Cotman et al., 2007). If PA intensity is important for central growth factor expression to promote hippocampal neurogenesis in the DS brain, it is possible that our adults with DS did not achieve a sufficiently high PA intensity to show any impact on hippocampal volume. Instead, other benefits of PA may be at play that target more widespread neural benefits to promote better memory. Prior research from our group demonstrated a positive association between the proportion of time engaged in moderate PA and white matter microstructural integrity in several tracts connecting broad areas of the brain (Fleming et al., 2021). These findings supported the hypothesis that PA was associated with widespread, rather than focal, benefits to brain health in adults with DS. More research in this area is needed.

This study is not without its limitations. First, our limited understanding of physiological differences between the DS and neurotypical population underscores a lack of actigraphy cutoffs specific to DS. It is possible that the actigraph watch cutoffs used in this study underestimate PA intensity, as they are based on a neurotypical population (Freedson et al., 1998). If true, this may explain the non-significant relationship between PA engagement and hippocampal volume in this DS sample. Second, using wrist-worn accelerometers may have inflated the average daily step count and/or time in moderate intensity PA (Rowlands and Stiles, 2012). However, we selected a wrist-worn accelerometer due to increased wear compliance compared to hip-worn accelerometers and the algorithm used to interpret the PA data accounted for wrist wearing and can capture meaningful differences in PA (Trost et al., 2014). Third, compared to the larger ABC-DS participant pool, we were limited by a relatively small sample size to examine the effects of PA, which may be of small-effect size (Erickson et al., 2019b). Fourth, our sample consisted only of White individuals, limiting our generalizability to adults with DS of other racial and ethnic backgrounds. Finally, given the cross-sectional nature of this study, we cannot make any inferences regarding causality in the relationships between PA, memory function, and hippocampal volume. Just as it is possible that greater engagement in moderate PA may have effects on memory performance and hippocampal volume, it is similarly possible that individuals with greater memory capacity and hippocampal volume are those who choose to be more active in their everyday lives. Additional longitudinal work is required to determine the temporal causality of these relationships.

Despite these limitations, the present study also has several notable strengths. To our knowledge, this is the first study to demonstrate positive associations between PA engagement and memory function in midlife adults with DS. This highlights the role of PA as a modifiable lifestyle behavior to promote better cognitive aging, regardless of premorbid status. Further, we showed that the known relationship between hippocampal volume and memory function extends to a non-neurotypical population.

In all, we demonstrated that greater engagement in PA is related to better memory function in midlife DS adults, suggesting that PA should be promoted across non-neurotypical populations in addition to neurotypical aging adults. We further demonstrated that larger hippocampal volume is closely related to better memory function in a non-neurotypical sample, expanding our understanding of brain-cognition relationships. The strengths and limitations of our study pave the way for future work, particularly in understanding how the relationships between PA, memory, and the hippocampus may persist or change over time in DS. The longitudinal nature of the ABC-DS study will allow future work to evaluate those possible changes, and hopefully, clarify the relationships in DS. Expanding this work into larger non-neurotypical samples, even at a cross-sectional level, could also elucidate some of the mechanisms underlying the relationships between PA and memory or hippocampal volume. Until then, PA can and should be promoted as a positive habitual behavior to benefit memory function in the DS population at high risk for developing clinical AD later in life.

## Data Availability Statement

The data analyzed in this study was obtained from The National Institutes of Health (NIH) National Institute on Aging (NIA) Alzheimer’s Biomarkers Consortium—Down Syndrome (ABC-DS), the following licenses/restrictions apply: Applicants must complete and submit a data request form (ABC-DS Data Request Form) and review and sign the ABC-DS Data Use Agreement. Requests to access these datasets should be directed to ABC-DS, https://www.nia.nih.gov/research/abc-ds#data.

## Ethics Statement

The studies involving human participants were reviewed and approved by University of Pittsburgh Institutional Review Board and University of Wisconsin Institutional Review Board. The patients/participants provided their written informed consent to participate in this study.

## Author Contributions

The authors confirm contribution to the article as follows: Study conception and design: JP, SH, BH, BC, WK, and OO. Data collection: BP-G and VF. Analysis and interpretation of results: JP, CL, SH, and BH. Manuscript draft preparation: JP and SH. All authors contributed to the article and approved the submitted version.

## Funding

Funding for this manuscript was provided by the National Institutes of Health (U01 AB051406; U19AG068054; R01AG070028; U54 HD09025).

## Conflict of Interest

GE Healthcare holds a license agreement with the University of Pittsburgh based on technology co-invented by WK. GE Healthcare did not provide grant support for this study and had no role in the design or interpretation of results or preparation of this manuscript. The remaining authors declare that the research was conducted in the absence of any commercial or financial relationships that could be construed as a potential conflict of interest.

## Publisher’s Note

All claims expressed in this article are solely those of the authors and do not necessarily represent those of their affiliated organizations, or those of the publisher, the editors and the reviewers. Any product that may be evaluated in this article, or claim that may be made by its manufacturer, is not guaranteed or endorsed by the publisher.

## Footnotes

^1^ http://www.nia.nih.gov/research/abc-ds

^2^ http://surfer.nmr.mgh.harvard.edu/
